# Single-Cell Transcriptomic Analysis Reveals Multicellular Coordination and Signaling Rewiring During Fetal Goat Skeletal Muscle Development

**DOI:** 10.3390/ani16091370

**Published:** 2026-04-29

**Authors:** Shiyao Han, Shengcan Xie, Fenfen Jiang, Qianhui Zou, Tianle Li, Ahui Wang, Nan Wang, Chuzhao Lei, Young Tang

**Affiliations:** 1 Shaanxi Centre of Stem Cells Engineering & Technology, Key Laboratory of Livestock Biology, College of Veterinary Medicine, Northwest A&F University, Yangling 712100, China; hanshiyao@nwafu.edu.cn (S.H.); xieshengcan@nwafu.edu.cn (S.X.); ffj@nwafu.edu.cn (F.J.); 2020010670@nwafu.edu.cn (T.L.); 2024060400@nwsuaf.edu.cn (A.W.); 2 Guangdong Provincial Key Laboratory of Brain Connectome and Behavior, Brain Cognition and Brain Disease Institute (BCBDI), Shenzhen-Hong Kong Institute of Brain Science, Shenzhen Institutes of Advanced Technology (SIAT), Chinese Academy of Sciences (CAS), Shenzhen 518055, China; qh.zou@siat.ac.cn; 3 Key Laboratory of Animal Genetics, Breeding and Reproduction of Shaanxi Province, College of Animal Science and Technology, Northwest A&F University, Yangling 712100, China; wangnan1231223@163.com (N.W.); leichuzhao1118@126.com (C.L.)

**Keywords:** goat, muscle, single cell RNA-seq, skeletal muscle

## Abstract

Fetal skeletal muscle development involves coordinated interactions among myogenic, stromal, vascular, and immune compartments, yet the cellular and molecular programs guiding tissue maturation remain incompletely understood. To address this, we generated a high-resolution single-cell atlas of fetal female goat skeletal muscle and performed trajectory analysis, transcription factor activity profiling, and intercellular communication mapping. Unsupervised clustering identified RUNX2 mesenchymal progenitors, fibro-adipogenic progenitors (FAPs), myofibroblasts, endothelial cells, macrophages, differentiating myocytes, and mature skeletal muscle fibers, revealing a heterogeneous ecosystem in which stromal populations support myogenic progression and vascular and immune cells contribute to tissue organization. Pseudotime analysis traced a maturation continuum from differentiation-competent myocytes to contractile fibers, marked by sequential activation of extracellular matrix remodeling, cytoskeletal stabilization, and sarcomere assembly. KEGG and GO enrichment highlighted stage-specific engagement of ErbB, Hedgehog, and Hippo signaling, as well as cell cycle and ubiquitin-mediated proteolysis pathways, linking proliferation, differentiation, and structural maturation. Transcription factor profiling revealed early-stage proliferative and morphogenetically permissive states driven by E2F4/5, HMGA2, and HAND2, transitioning to late-stage differentiation, ECM remodeling, and tissue stabilization orchestrated by CEBPB, CREB3L1, ELK1, and E2F2. Cell–cell communication analysis showed a developmental redistribution of signaling authority, from ECM-driven, progenitor-centered networks to modular, structurally stabilized interactions. These findings define the cellular, transcriptional, and signaling framework orchestrating fetal skeletal muscle maturation.

## Simple Summary

Skeletal muscle development before birth plays a crucial role in determining how much muscle an animal can build after birth, which directly affects growth performance and meat production in livestock such as goats. However, the detailed cellular processes that shape muscle formation in ruminant species during pregnancy are still not fully understood. In this study, we used advanced single-cell technology to examine thousands of individual cells from fetal goat skeletal muscle, allowing us to identify the different cell types present and understand how they change over time. We found that muscle development is not driven by muscle cells alone. Instead, supportive cells that form connective tissue, blood vessels, and immune components actively interact with developing muscle cells to guide tissue formation. Early in development, cells are mainly focused on growth and building the structural framework of the tissue. Later, the system shifts toward strengthening and stabilizing muscle fibers so they can perform their contractile function. These findings provide a clearer picture of how muscle is formed before birth in goats and offer valuable knowledge that may help improve breeding strategies and enhance muscle growth potential and production efficiency in livestock.

## 1. Introduction

Skeletal muscle formation is a highly coordinated and tightly regulated process during mammalian embryonic development [1]. Classical developmental studies have established that skeletal muscle originates from the paraxial mesoderm, where myogenic progenitors undergo lineage commitment, differentiation, and fusion under the control of Pax3, Pax7 [2], and a hierarchy of myogenic regulatory factors (MRFs), ultimately giving rise to mature multinucleated myofibers [3,4,5]. This core myogenic program is highly conserved across mammalian species and provides a fundamental framework for understanding muscle development [6].

Increasing evidence indicates that skeletal muscle development is not driven solely by the intrinsic program of myogenic cells [7]. Developing muscle tissue contains multiple non-myogenic cell types, including fibroblasts, fibro-adipogenic progenitors (FAPs), endothelial cells, immune cells, and other stromal populations [8,9]. These cells are present from early developmental stages and influence myogenic cell proliferation and differentiation through extracellular matrix remodeling, tissue organization, and paracrine signaling [10,11]. Thus, skeletal muscle formation should be considered a multicellular process in which coordinated interactions among diverse cell types are essential for proper tissue assembly [12].

Bulk RNA sequencing has provided valuable insights into global transcriptional changes during muscle development but is inherently limited by the mixture of heterogeneous cell populations [13]. The advent of single-cell RNA sequencing (scRNA-seq) has enabled transcriptomic profiling at single-cell resolution, allowing complex tissues to be dissected into their constituent cell types [14]. This approach has been widely applied to adult skeletal muscle and injury-induced regeneration models, revealing cellular heterogeneity and cell–cell interactions that underlie muscle homeostasis [15]. In contrast, scRNA-seq studies of embryonic skeletal muscle, particularly in ruminant species, remain relatively limited.

Ruminant species are characterized by longer gestation periods, during which skeletal muscle development unfolds over an extended temporal window [16]. This feature provides an advantageous system for resolving dynamic changes in cellular states during myogenesis [17]. Recent single-cell studies in species such as pigs and cattle have begun to uncover transcriptional features of embryonic muscle development and suggest that, despite a conserved myogenic framework, species-specific differences in cellular composition and regulatory programs may exist [17,18].

Goats are an economically important livestock species, and embryonic skeletal muscle development plays a critical role in determining postnatal muscle growth potential [19]. Previous studies in goats have primarily relied on bulk transcriptomic analyses or in vitro culture systems to investigate signaling pathways involved in myogenic regulation, including Wnt, PI3K–Akt, and MAPK pathways [20]. However, a comprehensive characterization of cell-type composition and transcriptional states in embryonic goat skeletal muscle is still lacking. In this study, we applied single-cell RNA sequencing to embryonic goat skeletal muscle to systematically characterize its cellular landscape and the transcriptional features of major cell populations, providing new insights into cellular heterogeneity and potential regulatory mechanisms underlying skeletal muscle development in ruminant species [20,21].

## 2. Materials and Methods

### 2.1. Experimental Model and Data Availability

A total of 2 biological samples were collected, including one fetal goat at embryonic day 90 (E90) and one fetal goat at embryonic day 136 (E136). The fetuses were of mixed sex and obtained from pregnant dairy goats maintained under standard feeding conditions. Muscle tissues were consistently collected from the same anatomical region (longissimus dorsi) to minimize variability. Single-cell RNA sequencing data for goat muscle (E90 and E136) are available in the China National Center for Bioinformation (CNCB) database under accession number CNCB: OMIX015485.

### 2.2. Ethics Statement

All animal experimental procedures were performed in accordance with the National Research Council’s Guide for the Care and Use of Laboratory Animals and were approved by the Institutional Animal Care and Use Committee (IACUC) of Northwest A&F University (protocol code: IACUC2024-1204, date of approval: 10 December 2024).

### 2.3. Fetal Goat Muscle Tissue Acquisition

Donor does raised under natural conditions were monitored daily for estrus to confirm pregnancy. Artificial insemination was performed once at the onset of estrus. Uteri were collected from pregnant does at a local slaughterhouse and transported to a sterile laboratory for disinfection. For each developmental stage (E90 and E136), one fetus was obtained from one pregnant doe. The does were anesthetized with pentobarbital, and fetuses were delivered by cesarean section. Embryos were then dissected, and longissimus dorsi muscle (skeletal muscle) tissues were isolated [22,23,24,25]. To minimize tissue heterogeneity, all samples were consistently collected from the same anatomical region (longissimus dorsi). In addition, single-cell clustering and marker gene expression analyses were performed post hoc to confirm the absence of significant non-muscle tissue contamination. [22,23,24,25]. All animal experiments were conducted in accordance with relevant guidelines and regulations and were approved by the institutional animal care and use committee. E90 represents a mid-gestation stage characterized by active myogenic differentiation and expansion of stromal populations, whereas E136 corresponds to a late gestational stage marked by structural maturation of muscle fibers and establishment of tissue architecture. These two stages were selected to capture the key transition from proliferative to maturation phases during fetal skeletal muscle development in goats.

### 2.4. Single-Cell Suspension Preparation of Goat Muscle

To collect goat muscle were dissected under a stereomicroscope and transferred to a 1.5 mL tube containing a digestion mix of 1 mg/mL Collagenase IV, 1 mg/mL Hyaluronidase, 0.25% Trypsin, and 1 mg/mL DNase I, followed by incubation at 37 °C for 30 min. After digestion, serum was added to neutralize the enzymes [14,26,27]. The cell suspension was pipetted repeatedly, filtered through a 40 μm cell strainer, and resuspended in 0.1% serum/PBS to prepare single-cell suspensions for 10× Genomics single-cell RNA-seq [23,24].

### 2.5. Single-Cell RNA Sequencing

Single-cell suspensions with viability exceeding 80% were adjusted to a concentration of 700–1200 cells/μL. Libraries were generated using the MobiCube^®^ High Throughput Single Cell 3′ RNA-Seq Kit (v2.1) according to the manufacturer’s instructions. Single cells were encapsulated into droplets using a microfluidic platform, enabling barcoding of individual transcripts via gel beads carrying unique molecular identifiers. Following reverse transcription, barcoded cDNA was amplified and sequencing libraries were constructed using the corresponding 3′ indexing reagents. Libraries were sequenced on an Illumina NovaSeq platform (San Diego, CA, USA) with paired-end 150-bp reads [28,29,30].

### 2.6. RNA-Seq Library Preparation and Sequencing

Total RNA was extracted using the miRNeasy Mini Kit (Qiagen, Hilden, Germany), and RNA integrity was assessed with an Agilent 2100 Bioanalyzer (Santa Clara, CA, USA). Library preparation was performed following standard protocols, including RNA fragmentation, first- and second-strand cDNA synthesis, end repair, adaptor ligation, and PCR amplification [31,32]. Final libraries were purified using AMPure XP beads and quality-checked prior to sequencing. Paired-end sequencing (150 bp) was performed on an Illumina NovaSeq 6000 platform, generating approximately 50 million reads per sample [33].

### 2.7. Single-Cell RNA-Seq Data Processing

Raw sequencing data were processed using Cell Ranger (v3.1, 10× Genomics). Reads were aligned to the goat reference genome (Capra hircus, ARS1 assembly), following the Cell Ranger recommended pipeline. The reference genome and gene annotation files were obtained from Ensembl (release 98). Filtered feature–barcode matrices generated by Cell Ranger were used for all downstream analyses, including cell clustering, differential expression, and pathway enrichment [27,34,35].

### 2.8. Cell Clustering and Cell Type Annotation

Single-cell data were analyzed using Seurat (v5.2.0). Cells expressing fewer than 300 genes or exhibiting mitochondrial gene expression exceeding 10% were excluded. Genes detected in fewer than three cells were also removed [29,36]. After normalization, the top 2000 highly variable genes were selected for downstream analysis [37]. Batch effects were corrected using Harmony, and the integrated data were subjected to dimensionality reduction and clustering. Cell types were annotated manually based on the expression of established marker genes [38,39].

### 2.9. Differential Gene Expression and Functional Enrichment Analysis

Differentially expressed genes (DEGs) were identified for each cell type using the Wilcoxon rank-sum test. Genes with Bonferroni-adjusted *p* values < 0.05 were considered significant. Functional enrichment analyses, including Gene Ontology (GO) and KEGG pathway analysis, were performed using the clusterProfiler package (v4.2.1) to identify overrepresented biological processes and signaling pathways [40,41,42,43,44].

### 2.10. Transcriptional Regulatory Network Analysis

Gene regulatory network inference was performed using pySCENIC (v0.11.2). Co-expression modules between transcription factors and candidate target genes were first identified based on expression correlations [45]. These modules were refined through motif enrichment analysis to define regulons, consisting of transcription factors and their putative direct targets [46]. Regulon activity was subsequently quantified at the single-cell level, and transcription factors with high confidence and regulatory strength were retained for further analysis [47,48].

### 2.11. Pseudotime and Trajectory Analysis

Cellular differentiation trajectories were reconstructed using Monocle3 [49]. Cells were ordered along pseudotime following dimensionality reduction with UMAP [50]. Based on canonical marker gene expression and known developmental relationships, progenitor-like cells were designated as the root of the trajectory [51,52]. Genes associated with dynamic expression changes along pseudotime were identified using the graph_test function, enabling characterization of transcriptional programs underlying developmental progression [53].

### 2.12. Cell–Cell Communication Analysis

To characterize intercellular signaling, cell–cell communication was examined using CellChat (v1.6.1) [54]. Putative signaling interactions were inferred by mapping annotated cell populations to a reference compendium of experimentally supported ligand–receptor pairs [55]. For each interaction, communication likelihood and signaling intensity were computed [56]. Both the global communication landscape and selected signaling pathways were subsequently visualized.

## 3. Results

### 3.1. Single-Cell Atlas Defines Stromal–Myogenic Organization in Fetal Female Goat Skeletal Muscle

**Figure 1:**
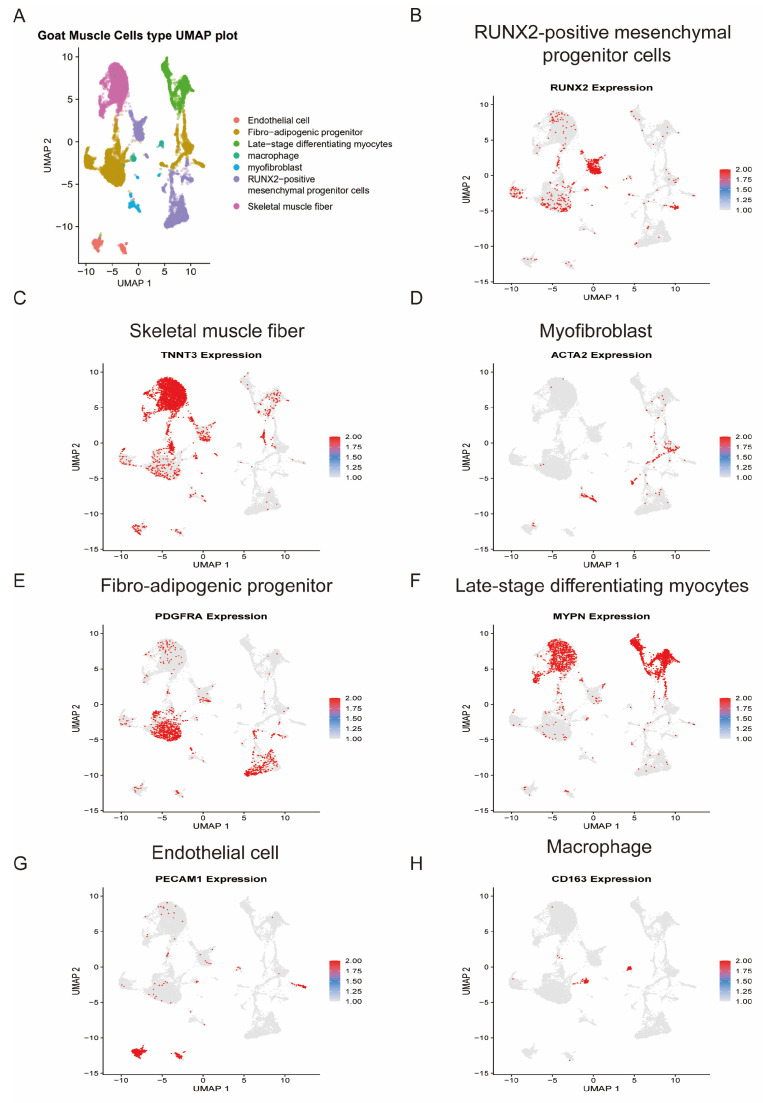
Single-cell transcriptomic landscape of fetal female goat skeletal muscle. (**A**) UMAP visualization of major cell populations. (**B**) RUNX2 marks mesenchymal progenitor cells. (**C**) TNNT3 identifies skeletal muscle fibers. (**D**) ACTA2 defines myofibroblasts. (**E**) PDGFRA identifies fibro-adipogenic progenitors. (**F**) MYPN marks late-stage differentiating myocytes. (**G**) PECAM1 defines endothelial cells. (**H**) CD163 identifies macrophages.

To comprehensively characterize the cellular landscape of fetal female goat skeletal muscle, we performed single-cell RNA sequencing followed by UMAP dimensional reduction (Figure 1A, Table S1). Unsupervised clustering resolved transcriptionally distinct populations corresponding to major myogenic, stromal, vascular, and immune compartments, consistent with the cellular architecture reported in mammalian fetal muscle development [2,57].

Based on canonical marker gene expression, we annotated RUNX2-positive mesenchymal progenitor cells, skeletal muscle fibers, late-stage differentiating myocytes, myofibroblasts, fibro-adipogenic progenitors (FAPs), endothelial cells, and macrophages (Figure 1B–H).

RUNX2 expression was specifically enriched in a mesenchymal progenitor cluster (Figure 1B), with concurrent expression of DCN and COL1A1 (Supplementary Figure S1E), suggesting the presence of multipotent stromal precursors within the developing muscle. RUNX2 has been implicated in mesenchymal lineage specification and skeletal progenitor regulation [58], and its enrichment here indicates an active progenitor pool contributing to stromal and connective tissue components during fetal development.

The myogenic lineage was resolved into at least two transcriptionally distinct states. Skeletal muscle fibers showed robust expression of TNNT3 (Figure 1C), a marker of fast-twitch contractile apparatus assembly [59], along with the expression of ACTA1 and CSRP3 (Supplementary Figure S1A), supporting their mature contractile phenotype. indicating structurally mature myofibers. In contrast, late-stage differentiating myocytes were characterized by strong MYPN expression (Figure 1F), a sarcomeric protein associated with myofibrillogenesis and myocyte maturation [60], along with the expression of MYH3 (Supplementary Figure S1B). The separation of these two clusters suggests progressive maturation along the myogenic continuum, consistent with staged fetal myogenesis described in mammalian systems [61].

Stromal populations were clearly resolved. Myofibroblasts expressed ACTA2 (Figure 1D), VWF and KDR (Supplementary Figure S1F), reflecting contractile and extracellular matrix (ECM)-remodeling properties typical of interstitial fibroblasts during tissue morphogenesis [62]. Fibro-adipogenic progenitors (FAPs), defined by PDGFRA expression (Figure 1E) and further supported by the expression of CD34 and COL3A1 (Supplementary Figure S1G), represent a well-established interstitial progenitor population that supports myogenesis and modulates regenerative and developmental environments [63]. The coexistence of RUNX2 mesenchymal progenitors and PDGFRA^+^ FAPs highlights stromal heterogeneity within fetal muscle.

In addition, vascular and immune compartments were evident. Endothelial cells expressed PECAM1 (Figure 1G) and TAGLN (Supplementary Figure S1C), consistent with active vascularization, which is tightly coordinated with myogenesis during development [64]. Macrophages expressing CD163 (Figure 1H) were also detected, with concurrent expression of CSF1R (Supplementary Figure S1D), further supporting their identity and suggesting immune participation in developmental remodeling, a phenomenon increasingly recognized in embryonic tissue maturation [65].

This single-cell atlas reveals that fetal female goat skeletal muscle is composed of a structured yet heterogeneous cellular ecosystem in which stromal progenitors, differentiating myocytes, vascular cells, and immune cells coexist. The clear segregation of myogenic maturation states together with multiple stromal subsets provides a cellular framework for subsequent analyses of intercellular communication and transcriptional regulation during fetal muscle development. Together, these results demonstrate that fetal goat skeletal muscle is composed of a heterogeneous cellular ecosystem, including myogenic, stromal, vascular, and immune cell populations, providing a foundation for subsequent analyses of intercellular communication and regulatory mechanisms.

### 3.2. Cross-Species Germ Cell Developmental Trajectories Reveal Conserved Programs and Divergent Meiotic Timing

**Figure 2:**
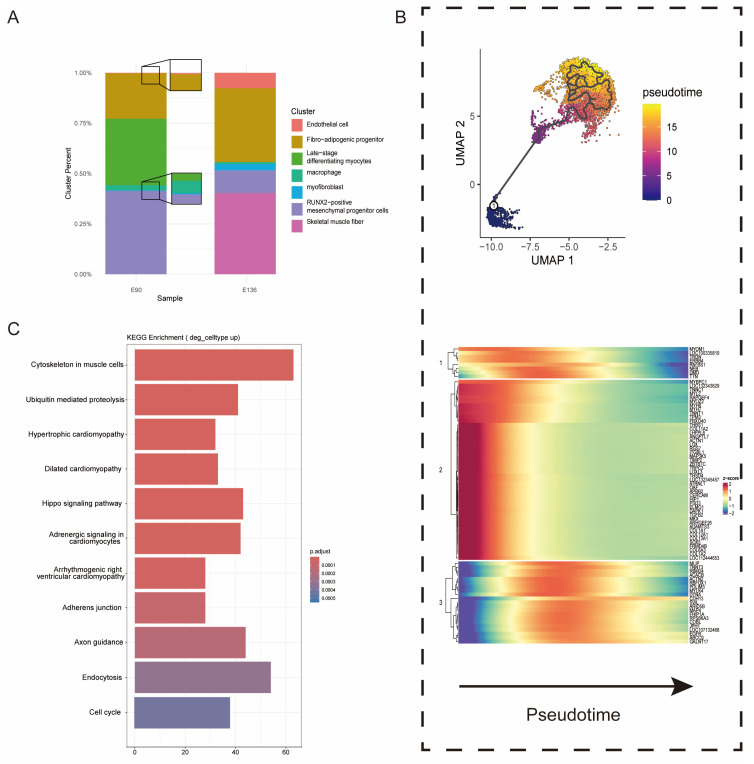
Cellular Composition, Pseudotime Dynamics, and Functional Programs during Fetal Goat Skeletal Muscle Development (**A**) Major cell type composition of fetal goat skeletal muscle at early and late developmental stages. (**B**) (**Top**): Pseudotime trajectories of skeletal muscle cells in goats. (**Bottom**): Heatmap for gene expression levels along the pseudotime trajectories. (**C**) KEGG analysis based on these DEGs.

To investigate skeletal muscle development, we first quantified cell population dynamics across two defined developmental stages: E90 (early stage) and E136 (late stage) (Figure 2A). Both stages contained major cell types, including endothelial cells, fibro-adipogenic progenitors (FAPs), late-stage differentiating myocytes, macrophages, myofibroblasts, RUNX2-positive mesenchymal progenitor cells, and skeletal muscle fibers, but their relative abundances changed markedly over time. At E90 (early stage), late-stage differentiating myocytes comprised 42% of cells, whereas mature skeletal muscle fibers accounted for only 18%, indicating ongoing active differentiation. FAPs and RUNX2-positive mesenchymal progenitors maintained substantial proportions, highlighting the critical support of stromal cells during early myogenesis. By E136 (late stage), skeletal muscle fibers increased to 46%, while late-stage differentiating myocytes decreased to 28%, reflecting a population-level transition from differentiating to terminally mature myocytes. Endothelial cells and macrophages remained relatively stable, suggesting continuous involvement of vascular and immune components in tissue organization and homeostasis.

To further resolve the maturation trajectory, we performed pseudotime analysis focusing on myogenic lineage cells, including late-stage differentiating myocytes and skeletal muscle fibers (Figure 2B, Table S2). Based on established myogenic differentiation hierarchies, late-stage differentiating myocytes were defined as the root of the trajectory, as they represent a transitional state preceding terminal myofiber maturation [2]. This trajectory structure is consistent with canonical models of skeletal muscle development, in which myocytes progressively differentiate and fuse to form mature contractile fibers. This analysis revealed a continuous developmental progression, with cells transitioning from a differentiation-competent state to terminal contractile specialization. Early pseudotime was characterized by high expression of genes involved in extracellular matrix remodeling and structural plasticity, including THBS1, COL1A1 [66], COL6A3 [67], LOX [68], and TGFB2 [69]. GO enrichment analysis confirmed significant terms related to ECM organization, collagen fibril organization, and cell adhesion, indicating that early-stage myocytes retain remodeling capacity. As pseudotime progressed, genes regulating cytoskeletal stabilization and sarcomere assembly (RBM24, PDLIM3, DTNA, SVIL) were upregulated, culminating in peak expression of canonical contractile genes MYH6, MYH7, MYOM1, NEB, TTN, MYBPC1, TNNC1 [69], and ACTN2 at terminal pseudotime. GO analysis of late-stage genes highlighted enrichment for sarcomere organization, myofibril assembly, muscle contraction, and actin filament-based processes, reflecting functional maturation. We further compared gene expression profiles between E90 and E136 skeletal muscle cells and performed KEGG pathway enrichment analysis on upregulated genes (Figure 2C). This revealed pronounced activation of pathways central to muscle growth and differentiation, including ErbB, Hedgehog, and Hippo signaling, highlighting coordinated control of proliferation, lineage specification, and tissue expansion. Consistent with previous reports, ErbB signaling modulates myoblast proliferation and neuromuscular development [70,71]. Together, these results integrate population-level remodeling with trajectory-resolved transcriptional dynamics, outlining a coordinated program in which skeletal muscle progresses from an ECM-remodeling, differentiation-competent state at E90 toward cytoskeletal consolidation and functional contractile maturation at E136.

### 3.3. Stage-Specific Transcription Factor Analysis of Fetal Goat Skeletal Muscle

**Figure 3:**
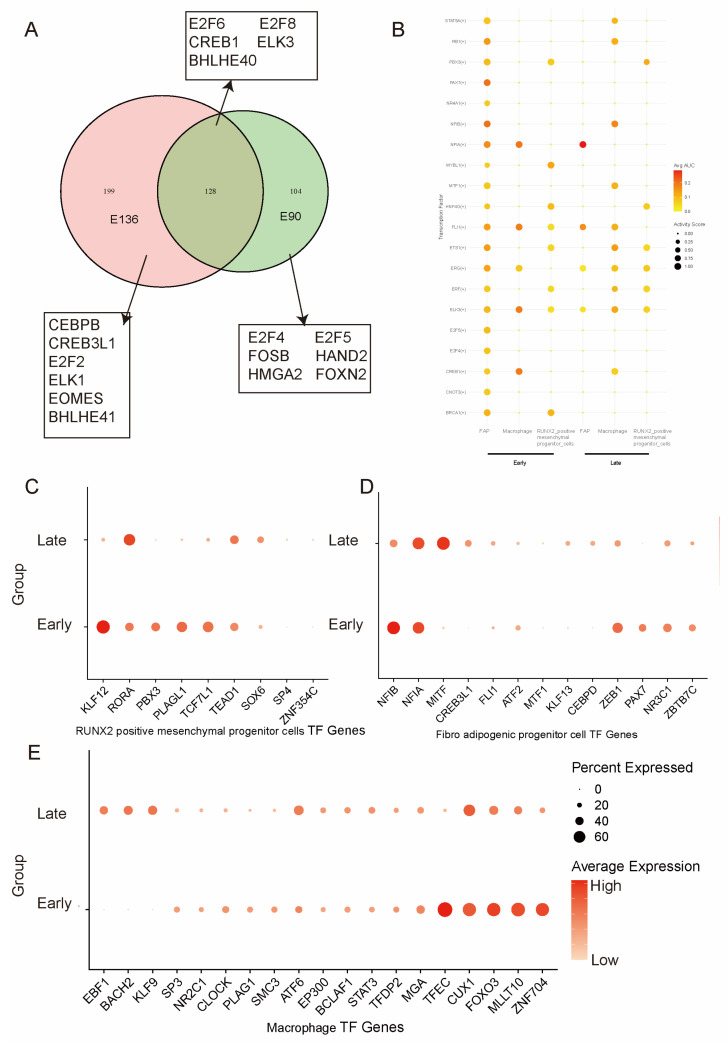
Dynamic regulon landscape across mesenchymal progenitors, FAPs, and macrophages during fetal goat muscle development. (**A**) Venn diagram showing the regulons overlapping in muscle cells of goats. (**B**) Comparative analysis of highly active transcription factors in RUNX2-positive mesenchymal progenitor cells, macrophages, and fibro-adipogenic progenitors across developmental stages. (**C**) Dot plot showing mean expression levels of representative TFs in RUNX2^+^ mesenchymal progenitors of goats. (**D**) Dot plot showing mean expression levels of representative TFs in fibro-adipogenic progenitor cells of goats. (**E**) Dot plot showing mean expression levels of representative TFs in macrophages of goats. For all dot plots (**C**–**E**), the percentage of cells expressing each gene is indicated by dot size, and expression levels are color-coded based on logarithm-scaled normalized counts.

To elucidate the regulatory architecture governing fetal goat skeletal muscle development, we systematically compared regulon activities between early and late developmental stages across stromal-associated cell populations, including RUNX2^+^ mesenchymal progenitors (Figure 3C), fibro-adipogenic progenitors (FAPs) (Figure 3D), and macrophages (Figure 3E). A global analysis of transcription factor activity was performed, followed by assessment of cell type-specific regulatory enrichment patterns (Figure 3B, Table S4).

A subset of transcription factors, including E2F6, E2F8, CREB1, ELK3, and BHLHE40, remained persistently active across both stages, constituting a conserved regulatory backbone. Given their established roles in integrating cell cycle progression with MAPK/cAMP signaling pathways, sustained activity of these factors suggests maintenance of basal proliferative competence and signaling responsiveness throughout fetal myogenesis. In contrast, early-stage muscle exhibited pronounced enrichment of E2F4, E2F5, HMGA2, HAND2, and FOSB regulons (Figure 3A, Table S3). Collectively, these transcriptional signatures define early fetal muscle as a proliferative and morphogenetically permissive state characterized by progenitor amplification and niche construction.

By contrast, late-stage muscle preferentially activated CEBPB, CREB3L1, E2F2, ELK1, and BHLHE41 regulons, reflecting a transition toward differentiation, extracellular matrix remodeling, and tissue stabilization. Notably, the early predominance of E2F4/5 is supplanted by E2F2 enrichment at the late stage, indicating subtype-specific remodeling of the cell cycle regulatory axis and suggesting coordinated coupling of cell cycle withdrawal with lineage progression (Figure 3B).

At the level of individual cell populations, stromal and immune compartments exhibited pronounced stage-dependent transcriptional reconfiguration. RUNX2^+^ mesenchymal progenitors displayed elevated expression of KLF12, PBX3, PLAGL1, and TCF7L1 during early development, consistent with maintenance of an undifferentiated and proliferative stromal phenotype (Figure 3C, Table S5). Late-stage upregulation of RORA suggests progression toward a more lineage-restricted, structurally supportive mesenchymal identity potentially associated with tendon and osteogenic-like differentiation. FAPs similarly demonstrated early enrichment of NF1B, NF1A, ZEB1, and PAX7 [2], accompanied by NR3C1 and ZBTB7C expression, indicative of progenitor-like plasticity and potential myogenic competence. In late muscle, enhanced NF1A and MITF activity, together with sustained CREB3L1 and ZEB1 expression (Figure 3D, Table S5), supports commitment toward adipogenic and fibrogenic programs and active participation in extracellular matrix remodeling. Macrophages also underwent marked functional transition, shifting from early expression of TFEC, CUX1, FOXO3, MLLT10, and ZNF704—consistent with a progenitor-like, tissue-constructive state—to late enrichment of NF1A, MITF, EBF1, BACH2, and KLF9, indicative of a mature immune-regulatory phenotype contributing to tissue remodeling and homeostatic stabilization (Figure 3E, Table S5).

Collectively, these findings reveal coordinated, stage-dependent transcriptional reprogramming across mesenchymal and immune cell compartments during fetal skeletal muscle development. The progressive transition from a proliferative, morphogenetically plastic state to a differentiated and structurally stabilized state underscores the existence of a multicellular regulatory network orchestrating fetal muscle morphogenesis and microenvironmental maturation.

### 3.4. Developmental Rewiring of Intercellular Communication During Fetal Skeletal Muscle Maturation

**Figure 4:**
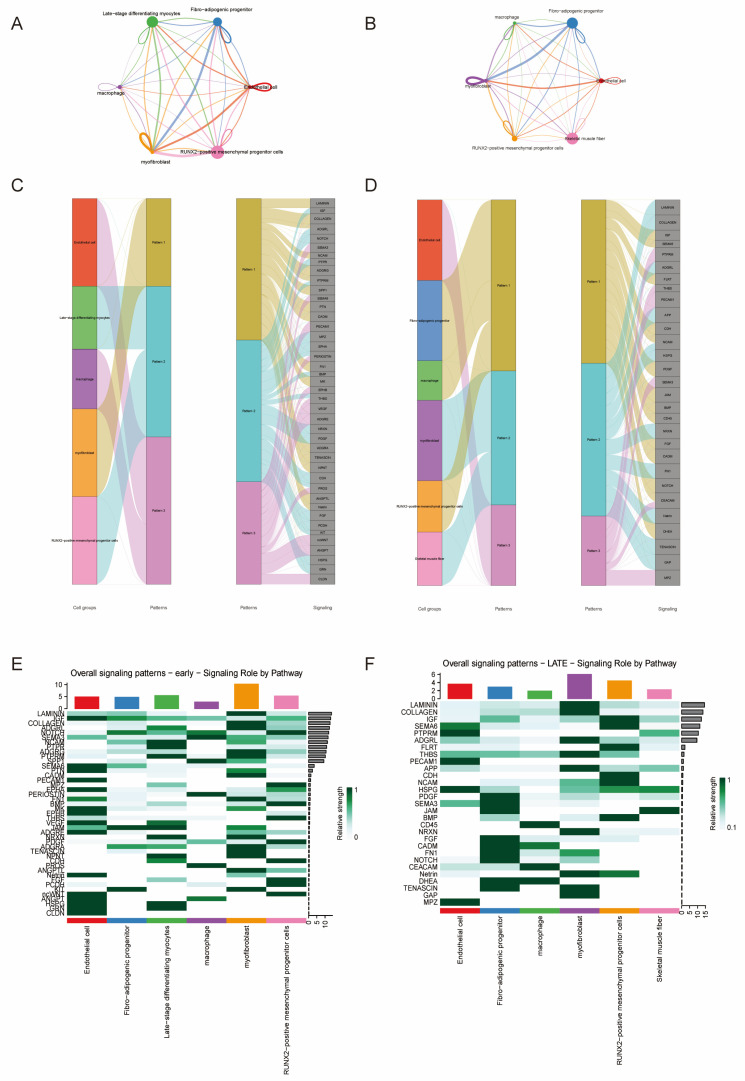
Developmental Remodeling of Cell–Cell Communication Networks in Fetal Goat Skeletal Muscle. (**A**) Overall cell–cell communication network in E90 goat muscle. (**B**) Overall cell–cell communication network in E136 goat muscle. (**C**) Incoming communication pattern analysis of signaling interactions in E90 goat muscle. (**D**) Incoming communication pattern analysis of signaling interactions in E136 goat muscle. (**E**) signaling_role_heatmap in E90 goat muscle. (**F**) signaling_role_heatmap in E136 goat muscle.

To define how intercellular communication evolves during fetal skeletal muscle development, we compared ligand–receptor interaction networks between E90 and E136 embryos using CellChat (Figure 4). This analysis revealed a stage-dependent rewiring of signaling topology, marking a transition from a matrix-constructive to a structurally stabilized microenvironment.

At E90, the network exhibited a highly interconnected architecture with extensive bidirectional signaling among RUNX2^+^ mesenchymal progenitors, fibroblasts, fibro-adipogenic progenitors (FAPs), endothelial cells, and differentiating myocytes (Figure 4A). Mesenchymal and fibroblast populations functioned as dominant signaling hubs enriched for extracellular matrix (ECM)-associated pathways (Figure 4C). Differentiating myocytes were also strongly embedded within the network, indicating active integration of stromal cues during early myogenic progression. These features are consistent with a dynamically expanding tissue state driven by stromal–myogenic crosstalk [72].

By E136, the network became more modular and hierarchically organized (Figure 4B). Myofibroblasts emerged as a central signaling hub, whereas skeletal muscle fibers displayed reduced outgoing interactions and more restricted signaling inputs (Figure 4D). FAPs and macrophages formed a distinct interaction module, suggesting enhanced immune–stromal coordination during later maturation. This architectural shift indicates that regulatory control progressively relocates from proliferative progenitors to specialized stromal compartments.

Incoming signaling pattern analysis further supported this transition. At E90, signaling inputs were broadly distributed and enriched for ECM and morphogen-related pathways (Figure 4C). In contrast, at E136, inputs became compartmentalized: myofibroblasts preferentially integrated ECM-remodeling signals, FAPs and macrophages were enriched for cytokine-related pathways (Figure 4D), and skeletal muscle fibers displayed a signaling profile associated with structural maintenance.

Pathway-level analysis highlighted a functional shift between stages. The E90 network was dominated by ECM and developmental pathways, including COLLAGEN, LAMININ, FN1, THBS, PERIOSTIN, NOTCH, and VEGF signaling, consistent with active matrix deposition and tissue expansion (Figure 4E). By E136, CDH, GAP junction, BMP, PDGF, SEMA3, and TENASCIN signaling were enhanced, reflecting strengthened cell adhesion (Figure 4F), intercellular connectivity, and structural stabilization.

Together, these findings demonstrate a developmental redistribution of signaling authority during fetal muscle maturation: early-stage muscle is characterized by expansive matrix-driven communication, whereas late-stage muscle adopts a compartmentalized and stabilization-oriented signaling architecture.

## 4. Discussion

This study establishes a high-resolution single-cell transcriptomic atlas of fetal goat skeletal muscle and systematically delineates the cellular basis by which myogenic cells, mesenchymal progenitors, vascular cells, and immune cells cooperatively shape tissue architecture during development. In contrast to the traditional view that fetal myogenesis proceeds as a linear progression of myogenic differentiation [2,57], our findings support a “multi-lineage coordinated assembly” model, in which myofiber maturation, mesenchymal scaffold construction, and microenvironmental signaling are tightly coupled in both temporal and spatial dimensions to jointly drive tissue formation and stabilization.

Within the myogenic lineage, the transition from late-differentiated myocytes to mature myofibers exhibits a clear transcriptional hierarchy, reflecting the progressive assembly of sarcomeres and contractile machinery, consistent with classical models of stepwise myofibrillogenesis in mammals [73]. Hedgehog signaling guides myogenic lineage commitment [74], and Hippo signaling regulates muscle mass and fiber growth via YAP/TAZ-mediated transcription [2,75,76]. Enrichment of the Cell cycle and Ubiquitin-mediated proteolysis pathways further underscores dynamic regulation of proliferative capacity and protein turnover, essential for fiber maturation and structural refinement [72,77]. Importantly, this maturation trajectory does not occur in isolation. RUNX2 mesenchymal progenitors, PDGFRA FAPs, and ACTA2 myofibroblasts [78] are closely associated with myogenic populations in transcriptional space, indicating that myofiber development is embedded within an active mesenchymal regulatory network. Previous studies have demonstrated that extracellular matrix (ECM) components, mechanical tension, and mesenchyme-derived growth factors critically influence myogenic fate decisions [79,80]. Our data further suggest that during early fetal stages in ruminants, the mesenchymal compartment may simultaneously provide structural support and integrate signaling cues, thereby establishing the foundation for subsequent myofiber alignment and tissue stability.

Notably, the mesenchymal compartment displays pronounced functional stratification. RUNX2 plays a central role in mesenchymal progenitor fate determination and osteo-connective lineage regulation [58], and its expression in fetal muscle suggests the presence of multipotent progenitors biased toward structural assembly. E2F4/5 are canonical mediators of proliferative cell cycle programs associated with expanding progenitor populations. HMGA2, a chromatin architectural factor highly expressed during embryogenesis, is implicated in stem/progenitor maintenance and developmental plasticity. Concurrent HAND2 activation indicates active mesenchymal patterning and structural framework establishment, consistent with the observed expansion of RUNX2^+^ stromal progenitors. CEBPB is closely linked to FAP maturation and metabolic reprogramming, whereas CREB3L1 directly regulates collagen synthesis and extracellular matrix gene networks. ELK1, a downstream effector of ERK/MAPK signaling, promotes differentiation-associated transcriptional cascades. PDGFRA FAPs have been shown to regulate myogenesis and maintain matrix homeostasis [81,82], whereas ACTA2^+^ myofibroblasts are closely associated with tissue tension maintenance and matrix remodeling [78]. This hierarchical organization implies that a foundational framework governing tissue mechanical properties and metabolic microenvironment is already established during fetal life. Given that the number of muscle fibers in ruminants is largely determined at birth [63], such structural preconfiguration may exert long-term effects on postnatal hypertrophic growth, fiber-type distribution, and adipogenic potential.

The integration of vascular and immune cells further highlights the systemic nature of fetal muscle development. Angiogenesis and myogenesis are tightly coupled during development [64] blood vessels not only provide metabolic support but also regulate myogenic activity through paracrine signaling. In parallel, fetal-derived macrophages contribute to cell clearance, tissue remodeling, and signaling modulation [65]. The detection of CD163^+^ macrophages in our dataset suggests that immune–developmental coupling mechanisms are conserved in ruminants.

From a developmental strategy perspective, the prolonged gestation period of ruminants and the prenatal determination of muscle fiber number render the fetal stage a critical window for establishing lifelong muscle growth potential [63]. The relatively abundant and functionally stratified mesenchymal system observed here suggests that large livestock species may adopt a “structural pre-assembly” strategy, establishing stable mechanical and signaling scaffolds before birth to support rapid postnatal weight gain. This strategy may represent a key developmental distinction between ruminants and model rodents [57].

Collectively, we propose a stage-dependent developmental model: early gestation features frequent interactions between myogenic and mesenchymal progenitors, whereas regulatory emphasis gradually shifts toward maintenance of the mesenchymal microenvironment as tissue architecture stabilizes, with mature myofibers primarily executing contractile function. This “developmental transfer of signaling dominance” may represent a fundamental mechanism underlying fetal skeletal muscle maturation.

Our findings further demonstrate that fetal muscle development involves structured interactions among myogenic cells, mesenchymal progenitors, fibro-adipogenic progenitors, vascular cells, and immune populations. The progressive transition from late-stage differentiating myocytes to mature myofibers is accompanied by a shift from extracellular matrix remodeling and proliferative plasticity toward sarcomere assembly and contractile specialization. In parallel, stromal and immune compartments undergo stage-dependent transcriptional reprogramming, moving from progenitor-enriched, morphogenetically active states to differentiated, matrix-stabilizing phenotypes. Importantly, intercellular communication analysis reveals a developmental rewiring of signaling networks, characterized by an early expansive, matrix-centered architecture and a later modular, stabilization-oriented configuration in which myofibroblasts and stromal subsets assume dominant regulatory roles. These findings support a model in which fetal skeletal muscle maturation is driven by a redistribution of signaling authority across cell types, integrating lineage progression with microenvironmental consolidation.

At single-cell resolution, this study provides a comprehensive view of the organizational principles of fetal skeletal muscle in ruminants, offering a new theoretical framework for understanding livestock muscle development. Future integration of cell–cell communication networks and causal regulatory analyses will help clarify lineage interdependencies and their decisive roles during developmental transitions.

## 5. Conclusions

This study establishes a comprehensive single-cell transcriptomic atlas of fetal goat skeletal muscle and provides a systems-level view of cellular composition, transcriptional regulation, and communication dynamics. These findings advance our understanding of mammalian muscle development and offer a conceptual foundation for future studies aimed at improving muscle growth potential and production traits in livestock.

## Data Availability

RNA-seq data were deposited in the China National Center for Bioinformation (CNCB) under accession code: OMIX015485.

## Supplementary Materials

The following supporting information can be downloaded at: https://www.mdpi.com/article/10.3390/ani16091370/s1, Table S1: goat muscle All cluster sig; Table S2: sig genes sorted by Pseudotime analysis; Table S3: Venn Regulons List; Table S4: Common High Active Regulons; Table S5: TF analysis. Figure S1: UMAP Visualization of Marker Genes in Different Cell Types.
