# Supplementary material for: Single-Cell Transcriptomic Analysis Reveals Multicellular Coordination and Signaling Rewiring During Fetal Goat Skeletal Muscle Development
Source: Animals (Basel). 2026 Apr 29;16(9):1370. doi: 10.3390/ani16091370 (PMC13162580; doi:10.3390/ani16091370)
Supplement: Supplementary file 1 [file animals-16-01370-s001.zip › animals-4214601-Figure S1.pdf]

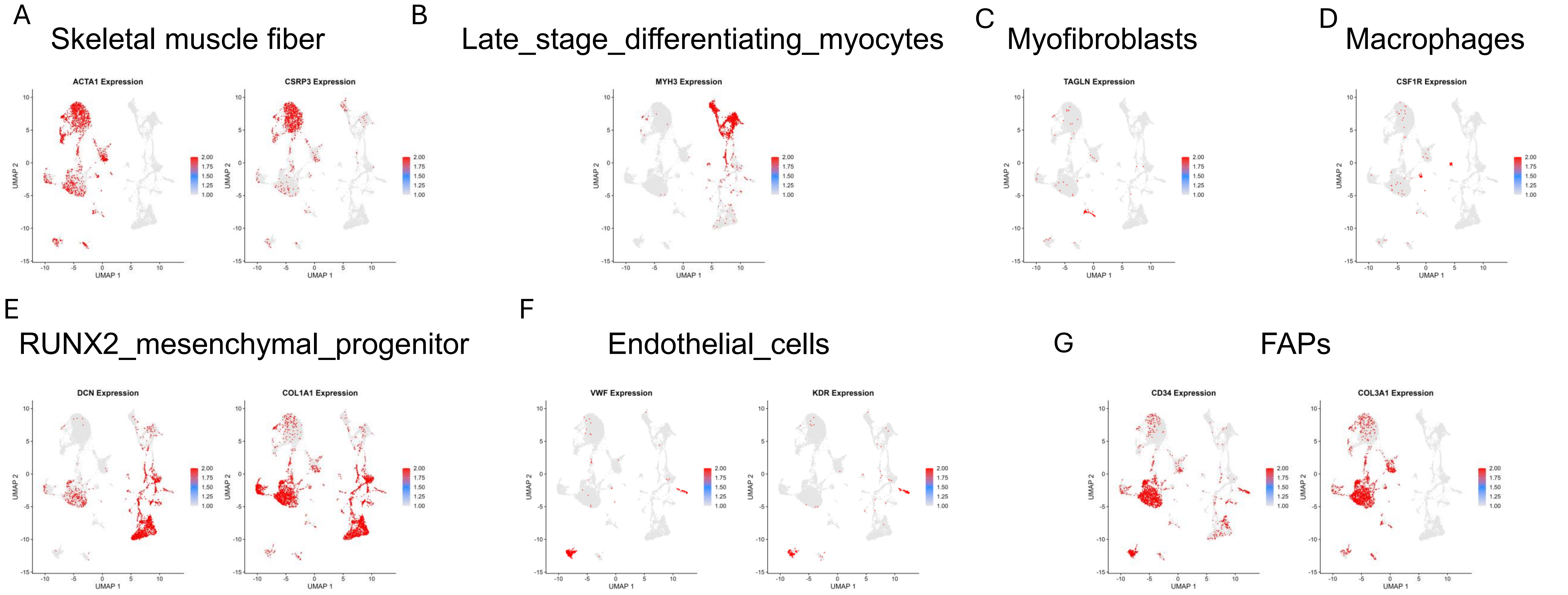
A
Skeletal muscle fiber
ACTA1 Expression
CSRP3 Expression
B
Late_stage_differentiating_myocytes
MYH3 Expression
C
Myofibroblasts
TAGLN Expression
D
Macrophages
CSF1R Expression
E
RUNX2_mesenchymal_progenitor
DCN Expression
COL1A1 Expression
F
Endothelial_cells
VWF Expression
KDR Expression
G
FAPs
CD34 Expression
COL3A1 Expression
UMAP 1
UMAP 2
2.00
1.75
1.50
1.25
1.00
